# Carbon Monoxide Poisoning Complicated by Ischemic Colitis: A Case Report and Literature Review

**DOI:** 10.7759/cureus.47669

**Published:** 2023-10-25

**Authors:** Ikuto Takeuchi, Youichi Yanagawa

**Affiliations:** 1 Acute Critical Care Medicine, Juntendo University Shizuoka Hospital, Izunokuni, JPN

**Keywords:** wrist cut, ischemic heart, cerebral ischemia, carbon monoxide poisoning, ischemic colitis

## Abstract

A 59-year-old male was found unconscious in a car filled with smoke. On arrival, he was in a semi-comatose state with hemorrhagic shock due to deep lacerations on his wrist. His carboxyhemoglobin level was 16.6%. Electrocardiography showed ST segment elevation at the precordial leads with troponin T positivity. Magnetic resonance imaging showed spotty ischemic lesions in his brain. He was treated with 100% oxygen by mechanical ventilation; however, he also developed acute respiratory distress syndrome due to an inhalation injury. His condition was complicated by bloody stools, which were judged to have been caused by ischemic colitis based on computed tomography and were managed by observation. After regaining consciousness and the improvement of the heart, lung, and bowel conditions, the patient was transported to a psychiatric hospital due to concerns regarding self-harm. Due to the small number of reported cases, the accumulation of similar cases of ischemic colitis after carbon monoxide (CO) poisoning is needed to clarify the characteristics of ischemic colitis after carbon monoxide poisoning.

## Introduction

Carbon monoxide (CO) poisoning accounts for thousands of deaths worldwide each year. The clinical effects can be diverse and include headache, dizziness, nausea, vomiting, syncope, seizures, coma, dysrhythmias, cardiac ischemia, and severe toxicity, which generally affect the nervous and cardiovascular systems [1]. The diagnosis can be elusive, as carboxyhemoglobin levels are not always correlated with the degree of poisoning [1].

Ischemic colitis results from an inadequate supply of oxygenated blood to any part of the colon and occurs as a result of non-occlusive ischemia due to small vessel disease in most cases [2]. Rarely, ischemic colitis occurs due to systemic hypotension or vascular occlusion due to atheromatous emboli, vasculitis, or vascular spasm [2]. Only four previous reports have concerned CO poisoning complicated by ischemic colitis [3-6]. Given the limited research on CO poisoning complicated by ischemic colitis, we herein report a rare case of colon, brain, and heart ischemia after CO poisoning.

## Case presentation

A 59-year-old male was found unconscious in a car filled with smoke. The patient was rescued by a firefighter who noticed the alarming concentration of CO using a detector inside the car. A physician-staffed helicopter was dispatched, and the patient was transported to our hospital after tracheal intubation (due to a semi-comatose state) with the provision of 100% oxygen. His history included hypertension, hyperuricemia, hyperlipidemia, an irritable colon, and thoracic ossification of the posterior longitudinal ligament, inducing walking disturbance, which had been surgically treated one year earlier but failed to allow him to regain normal walking ability. He had smoked 20 cigarettes per day for 30 years and drank 2 glasses of shochu (a Japanese distilled alcoholic beverage containing 25% alcohol) per day. Emergency physicians suspected suicide-related behavior because the patient had cuts on his wrist and was found in a car filled with smoke.

**Figure 1:**
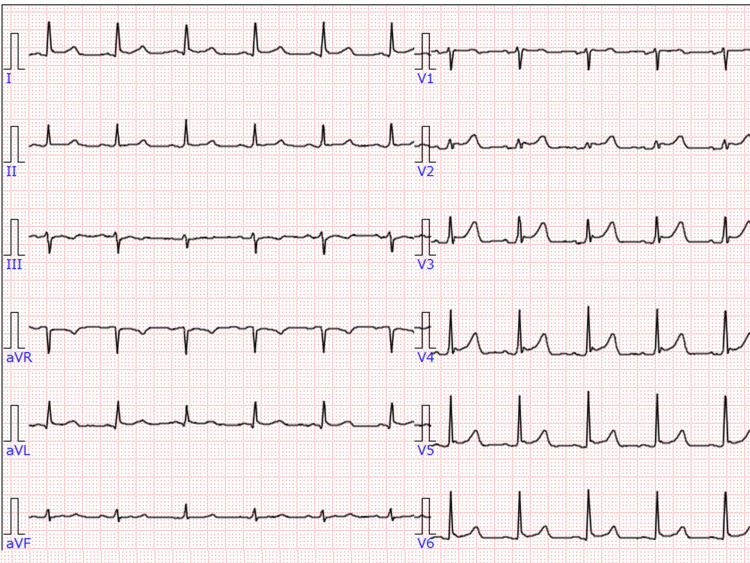
Electrocardiogram on arrival Electrocardiogram showed ST elevation at precordial leads.

On arrival, his vital signs were as follows: Glasgow Coma Scale, E2VTM6, blood pressure, 90/63 mmHg, and heart rate, 90 beats per minute. His face and hands were contaminated by soot. He also had lacerated wounds on the bilateral anterior wrists, which involved the superficial and deep finger tendons and the median nerve. The radial and ulnar arteries were intact. The results of a venous blood gas analysis were as follows: pH, 7.249; PaCO_2_, 53.0 mmHg; PaO_2_, 50.2 mmHg; $\mathrm{HCO_{3}^{-}}$, 22.4 mmol/L; base excess, −4.6 mmol/l, and carboxyhemoglobin, 16.6%. Bronchoscopy showed diffuse soot depositing in the bronchus. The electrocardiogram showed ST elevation at precordial leads (Figure 1).

The main findings of laboratory testing were leukocytosis, anemia, and signs of renal failure (Table 1).

**Table 1:** Results of a biochemical analysis on day 11

Variables	Level	Normal range
White blood cell count	25,000 /μL	3600–8900
Hemoglobin	10.7 g/dL	11.1–15.2
Platelet	19.7×10^4^/μL	15.3–34.6 × 10^4^
Total protein	5.1 g/dL	6.5–8.5
Albumin	2.9 g/dL	4–5.2
Aspartate aminotransferase	14 U/L	5–37
Alanine aminotransferase	9 U/L	6–43
Creatinine phosphokinase	77 U/L	47–200
Glucose	123 mg/dL	65–109
Blood urea nitrogen	26.5 mg/dL	9–21
Creatinine	2.47 mg/dL	0.5–0.8
Sodium	138 mEq/L	135–145
Potassium	4.4 mEq/L	3.5–5.0
C-reactive protein	0.60 mg/dL	Under 0.3
Troponin T	44 ng/mL	Under 0.014 ng/mL
Prothrombin time international normalized ratio	0.96	0.85–1.15
Activated partial thromboplastin time	25.0 seconds	70–120
Fibrin degrade product	4.6 μg/mL	Under 10

**Figure 2:**
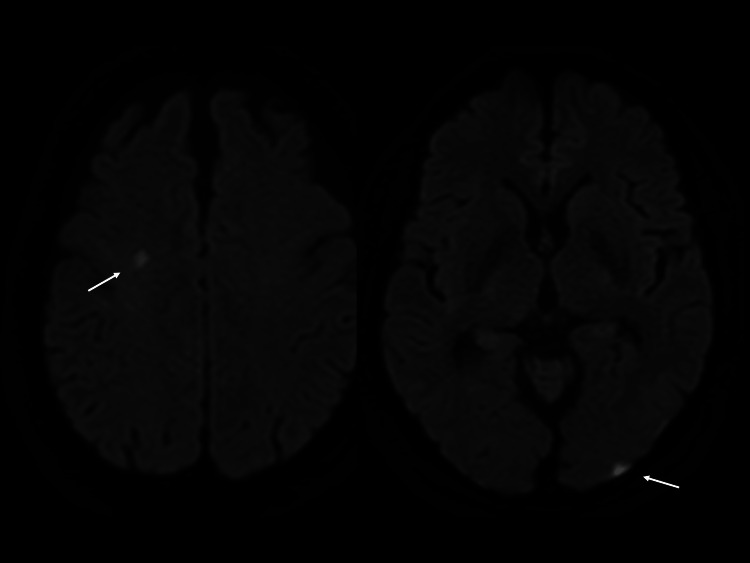
Diffusion-weighted axial magnetic resonance brain imaging on arrival Diffusion-weighted imaging showed two high intensity spots in the right centrum semiovale (left arrow) and left occipital cortex (right arrow).

Whole-body computed tomography (CT) that included the brain showed no significant ischemic lesions. However, diffusion-weighted magnetic resonance brain imaging showed two high-intensity spots in the right centrum semiovale and left occipital cortex (Figure 2).

**Figure 3:**
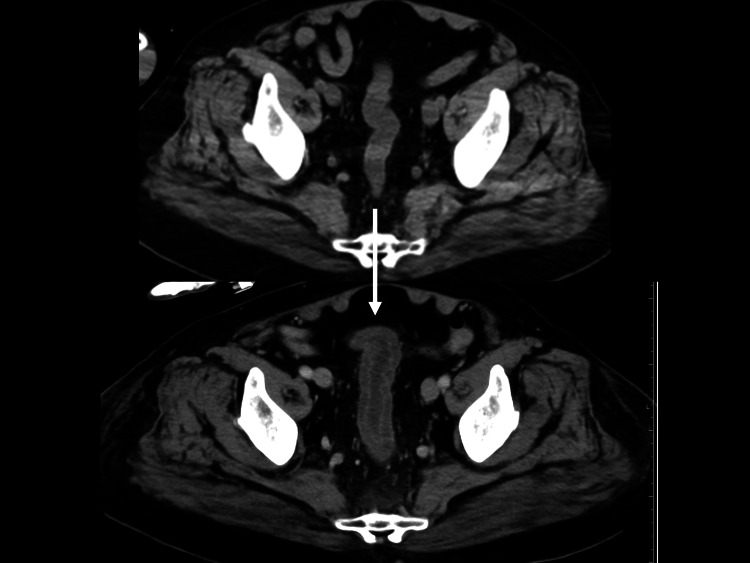
Abdominal computed tomography (CT) on arrival (upper) and day 3 (lower) CT showed thickening of the sigmoid and rectal colon, suggesting ischemic colitis. The arrow indicates the thickened sigmoid/rectal colon in the bottom image, with the top image showing the entity within normal limits.

He was diagnosed with CO poisoning, inhalation burn, cerebral and cardiac ischemia, deep wrist lacerated wounds, and hypovolemic shock with pre-renal renal failure. Surgery was performed to repair the severed tendons and median nerve, and the patient was admitted to the intensive care unit. The cerebral and cardiac ischemia in the heart and brain were managed conservatively. As his condition was complicated by acute respiratory distress syndrome on day 2, he received a tracheostomy on day 3. On the same day, he showed bloody stool; thus, an enhanced truncal CT was performed, which showed a thickening of the sigmoid and rectal colon (Figure 3).

It was considered that bloody stool had occurred as a complication of ischemic colitis, and a transfusion (4 units) was performed. This condition was also managed conservatively. His consciousness became clear on day 4, and mechanical ventilation was withdrawn on day 13 after the improvement of his respiratory function. The disappearance of his initial suicidal ideation induced by walking disturbance after the self-harm event was confirmed by psychiatrists and mental care for the patient was administered by them during hospitalization. He was finally transported to another hospital for rehabilitation for disuse atrophy, to improve the function of his bilateral hands, and for the treatment of psychiatric illness on day 28.

## Discussion

To the best of our knowledge, this is the first report of ischemia of the colon, brain, and heart together as complications after CO poisoning. Ischemia affecting the central nervous and cardiovascular systems commonly occurs after CO poisoning and may occur after ischemic stroke or ST-elevated myocardial infarction; in comparison, ischemic colitis is rare; thus, we focused on ischemic colitis in the present case [7,8].

Given the limited research on ischemic colitis after CO poisoning, we performed a Medline search in October 2022 to identify related articles using the keywords "CO poisoning" and "ischemic colitis," which identified four case reports [3-6]. Thus, the present case represents the fifth case report of ischemic colitis after CO poisoning (Table 2).

**Table 2:** Summary of case reports CT: computed tomography

Author	Sex	Age	Mechanism	CO-Hb (%)	Heart	Brain	Abdominal pain and other symptom	Onset of ischemic colitis	Diagnosis	Treatment	Others	Outcome
Weaver et al. [3]	Female	34	Car exhaust, suicide	23	T-wave inversions, increased troponin I	Coma	Yes, bloody stool	2 hours	Diffuse colonic mural thickening on CT, sigmoidoscopy showed an edematous friable pale mucosa from the rectum to distal sigmoid colon	Hyperbaric oxygen		Survival
Duenas-Laita et al. [4]	Female	87	Propane gas heater	19	ST segment depression	Semicoma	Yes, bloody stool	30 minutes	Colonoscopy showed the mucosa with edema, erythema, submucosal hemorrhage, and superficial longitudinal ulcerations in a 10 cm portion of the sigmoid colon	Oxygen		Survival
Balzan et al. [5]	Male	65	Butane gas water heater	45	ST segment depression	Coma	No	More than 14 hours	X-ray at autopsy showed widespread small bowel distension, ischemic necrosis of all abdominal organs including the liver, small and large bowel	Oxygen, mechanical ventilation	Lung edema, hypotension, liver/heart/brain/intestine necrosis by autopsy	Death
Watson et al. [6]	Female	24	Gas water heater	34	No	Coma	Yes	24 hours later	On laparotomy the bowel appeared blue but viable	Oxygen	Hepatic and renal failure	Survival
Present	Male	59	Smoke in the car, suicide	16	ST segment elevation, increased troponin T	Coma, high-intensity spots in right central semiovale and left occipital cortex	No, bloody stool	3 days	CT showed thickening of the sigmoid and rectal colon	Oxygen, mechanical ventilation	Anemia, renal failure	Survival

Our literature review revealed that all individuals with ischemic colitis due to CO poisoning initially showed unconsciousness with more than 16% carboxyhemoglobin and had delayed clinical features of ischemic colitis. The clinical features of ischemic colitis after CO poisoning in that study included bloody stool and thickening of the colon on CT. The present case only showed signs of ischemic colitis after more than one day; thus, in addition to CO poisoning, the complications of acute respiratory distress syndrome, anemia, and hypotension due to the patient’s bilateral wrist lacerations and/or renal failure may have contributed to the development of delayed ischemic colitis [2]. Only one patient received negative laparotomy for ischemic colitis before the era in which CT or colonoscopy was used for the diagnosis of acute abdomen. One of the patients died due to multiple organ ischemia, including ischemic colitis, which had been induced by severe CO poisoning.

As there have only been five case reports describing ischemic colitis after CO poisoning, the further accumulation of similar cases is needed to clarify the characteristics of ischemic colitis after CO poisoning.

## Conclusions

To the best of our knowledge, this is the first report of combined ischemia of the colon, brain, and heart as complications after CO poisoning. Physicians should be alert for the rare and potentially deadly complication of ischemic colitis when managing patients with severe CO poisoning.
